# Gatekeeper role of gastroenterologists and surgeons in recognising and discussing familial colorectal cancer

**DOI:** 10.1007/s10689-015-9861-5

**Published:** 2015-12-19

**Authors:** Kirsten F. L. Douma, Evelien Dekker, Ellen M. A. Smets, Cora M. Aalfs

**Affiliations:** Department of Medical Psychology, Academic Medical Center/University of Amsterdam, P.O. Box 22660, 1100 DD Amsterdam, The Netherlands; Department of Gastroenterology and Hepatology, Academic Medical Center, Amsterdam, The Netherlands; Department of Clinical Genetics, Academic Medical Center, Amsterdam, The Netherlands

**Keywords:** Hereditary colorectal neoplasms, Genetic testing, Gastroenterology, Risk assessment, Health communication

## Abstract

This study aimed to gain insight into the gatekeeper role of surgeons and gastroenterologists (including residents) during a first consultation at a tertiary gastro-intestinal centre regarding referral for genetic counselling, and to test the feasibility of a checklist for indications for referral. Consecutive patients were invited *before* and *after* introduction of a checklist, to complete a questionnaire assessing their perception of discussing cancer genetic topics. Initial consultations were audiotaped to assess the quality of this discussion by gastroenterologists and surgeons. Data on completeness of the checklist and referral were collected from medical files. No significant differences were found between the Before and After group regarding patients’ reports of discussing cancer in the family (77 %, n = 34 vs 89 %, n = 33, *p* = 0.16). In 28 % (n = 10) of the audiotaped consultations family history was adequately discussed, in 58 % (n = 21) it was considered inadequate and in 14 % (n = 5) of consultations it was not discussed at all. A checklist was present in 53 % (n = 27) of the medical files. Of these, 5 (19 %) were incomplete. Gastroenterologists and surgeons (in training) have difficulty in fulfilling their gatekeeper role of recognizing patients at familial risk for CRC. Although they often discuss familial cancer during the initial consultation, their exploration seems insufficient to reveal indications for referral for genetic counselling. Therefore, healthcare professionals should not only understand genetics and the importance of cancer family history, but also be effective in the communication of this subject to enable more adequate referral of patients for genetic counselling.

## Introduction

Worldwide, colorectal cancer (CRC) is among the five most common forms of cancer for both men and women. Although CRC is a common cause of cancer deaths, mortality can be reduced if cases are detected and treated early [WHO fact sheet http://www.who.int/mediacentre/factsheets/fs297/en/(November 2014)]. Approximately 10–30 % of colorectal cancers have a familial or hereditary nature [1, 2]. Health professionals (e.g. gastroenterologists and surgeons) function as gatekeepers, identifying patients at risk and providing them with initial information on heredity. Recognition of hereditary CRC syndromes (e.g. Lynch syndrome, familial adenomatous polyposis) is important to help identify high-risk patients and provide them with appropriate surveillance and surgical options. Recently published practice guidelines for genetic testing and management of hereditary gastrointestinal cancer syndromes stressed the importance of a standard minimal cancer family history assessment in gastrointestinal (GI) practice [3]. Despite the relatively high frequency of familial and hereditary CRC syndromes and the proven benefit of screening, referral for genetic counselling appears to be suboptimal leading to under-diagnosis of hereditary CRC [4]. Furthermore, referral is often patient-initiated leading to under-representation of patients with a lower education level, as they are less able to discuss their need for genetic counselling [5].

At the Gastro-Intestinal Center Amsterdam (GIOCA) expert centre, many patients with CRC are seen each week. Patients are recently diagnosed with CRC or referred to a tertiary centre for second opinion. An explorative investigation of the referral rates at this clinic to a clinical genetic centre for genetic counselling indicated suboptimal referral and showed that medical files had incomplete information or lacked information on family history. Therefore, a checklist was introduced for use by gastroenterologists and surgeons (in training) performing intake consultations at this clinic to: (1) improve the discussion on cancer in the family during the consultation, and (2) provide a tool for routine use of indications for referral from this clinic to a clinical genetic centre for genetic counselling.

However, the introduction of a checklist will only enhance appropriate referral to genetic services if the family history is adequately discussed with the patient. Although it is reported that in 80 % of the consultations, oncologists, surgeons and gastroenterologists discussed the cancer family history [6, 7], this discussion is often suboptimal. Even when clinicians did address the patient’s family history, an increased risk was only discussed in 57 % of those patients with an increased familial risk for CRC [6]. Adequate referral may be hampered by the clinician’s lack of knowledge; for example, Singh et al. [4] showed that, despite the presence of clear pathological criteria, Lynch syndrome remained under-recognised. Lack of experience may also explain limited referral, e.g. a study among internists showed that they experienced difficulties in discussing heredity and genetics with their patients as they lacked the training to do so [8]. Similarly, gastroenterologists and surgeons are not specifically trained in genetics and may lack the experience and skills to adequately discuss genetic issues with their patients.

This explorative study aimed to gain insight into the role of surgeons and gastroenterologists (in training) in referring patients for genetic counselling, and to test the feasibility of a supportive checklist. Specifically, this study investigated (1) whether introduction of a checklist increased surgeons’ and gastroenterologists’ discussion of family history and genetic counselling in a first consultation with patients with CRC, (2) whether the quality of the discussion of family history was sufficient to guarantee optimal referral for genetic counselling, (3) whether the checklist was filled in correctly, and (4) whether referrals to a clinical genetic centre were made when appropriate.

## Methods

### Study design and procedures

The Medical Ethical Committee of the Academic Medical Center gave an exemption for formal approval of the study.

#### Introduction of the checklist to increase discussion of family history and genetic counselling

At the Gastro-Intestinal Centre Amsterdam (GIOCA) expert centre all medical examinations take place on the same day. Patients are referred to this specialized clinic for a recently diagnosed CRC or referred as second opinion. They have an intake consultation with either a surgeon, surgeon in training or a gastroenterologist in training. The residents are in the final stage of their education and are therefore working independently, with supervision at distance. They have had only minor specific education in oncogenetic, apart from what they have learned during their medicine study. Subsequently, patients are discussed in a multidisciplinary team (including gastroenterologists, surgeons, radiotherapists, oncologists, radiologists, pathologists and specialized nurses) and receive a treatment plan (concerning surgery and (neo) adjuvant treatment) the same day. The clinical geneticists are located in the same hospital and regularly one of them is present at the multidisciplinary meeting.

We developed a checklist which surgeons and gastroenterologists (in training) could use during the intake consultation. The checklist was based on instruments already in use in other hospitals (9) and includes criteria for referral for genetic counselling derived from the Dutch CBO-guidelines (http://oncoline.nl/erfelijke-darmkanker), such as other family members with colon or endometrial cancer, young age of the patient (<50 years) and adenomatous or hyperplastic polyposis (see Table 1). If one of these criteria is present, referral for genetic counselling is indicated. A clinical geneticist (C.A.) introduced the checklist during a multidisciplinary meeting in which the checklist items were explained and the checklist was introduced as a helpful tool in daily practice. Gastroenterologists and surgeons were asked to use the tool to improve referral for genetic counselling. They were not informed that the checklist was part of the study as we wanted to observe a realistic setting. Studies have shown for video recordings, which is even more obtrusive than audio recording, that recording of consultations has little influence on the behaviour of either doctors or patients [10, 11]. The nurses, who were informed about the purpose of the study, were instructed to provide each gastroenterologist and surgeon (in training) with this checklist at each first consultation with a new patient. The researcher (K.D.) visited the GIOCA clinic every week to collect the questionnaires and coordinate the audio taping, At that time she also reminded the nurses to provide the checklist.

**Table 1:**
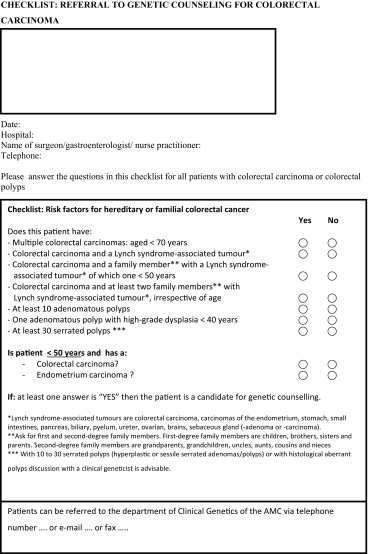
Translation of checklist

To investigate whether the introduction of this checklist increases discussion on family history and the possibility of referral for genetic counselling by surgeons and gastroenterologists (in training), an uncontrolled before and after study design was used.

Consecutive patients with a recent diagnosis of CRC were invited by the GIOCA nurses, after giving informed consent, to fill in a questionnaire immediately after their first consultation at the GIOCA outpatient clinic; one group was invited *before* introduction of the checklist (the Before group) and another group *after* introduction of the checklist (the After group). The first consultation was either with a gastroenterologist or with a surgeon (in training). The questionnaire investigated the patients’ perception of the degree to which family cancer history and genetic counselling was discussed during the consultation.

#### Quality of discussing family history

After the checklist was introduced to the clinicians, patients in the After group were asked by the researcher (K.D.) for their written informed consent to audiotape their consultation; the clinicians were also asked for their written informed consent. All clinicians, mostly residents, performing intake consultations at the clinic during the study period were included, none refused. Patients and clinicians were told that the study investigated patient-physician communication, without stressing the use of the checklist for discussion about cancer genetics, which was introduced some time before. Also, it was not mentioned to the clinicians that we focused on the discussion of family history and referral for genetic counselling. Neither patients nor clinicians were aware that the questionnaire, the checklist and the audiotapes were part of the same study.

#### Completeness of checklist and appropriate referral

Nurses scanned all checklists that were filled in by the clinicians and uploaded them to the electronic medical file. After completion of the study, the medical files of all participating patients were reviewed.

##### Sample size

Because of the explorative nature of the study, a large effect size (0.80) was assumed. Based on this effect size we calculated the needed sample size (G*Power 3.1.3) which showed that we needed 26 patients per group (Before and After). However, when taking into account a non-response of 30 % we aimed to invite 40 patients before introduction of the checklist and another 40 patients after introduction of the checklist, to fill in the questionnaire.

### Study sample

All five gastroenterologists in training, one surgeon, and three surgeons in training that were working at the clinic during the time of the study agreed to participate. None refused. Consecutive eligible patients with CRC referred to the GIOCA for a first consultation were invited to participate. Inclusion criteria were: a diagnosis of CRC, age ≥ 18 years, and adequate command of the Dutch language. Patients who were already known with hereditary cancer in the family were excluded.

### Measures

#### Patient questionnaire

The questionnaire asked for: (1) patients’ age and gender; (2) patients’ perception regarding whether cancer in the family was discussed during the consultation (yes/no) and who initiated this discussion (doctor/patient/don’t know); (3) when patients did discuss cancer in the family, they were asked what was discussed; whether the doctor asked: (a) who in the family has (had) cancer? (yes/no), (b) about cancer in first-degree family members (yes/no/don’t remember), (c) about cancer in second-degree family members (yes/no, don’t remember), (d) about the type of cancer of family members? (yes/no), and (e) about the age of the family members at the time of a cancer diagnosis? (yes/no); (4) finally, if relevant, patients were asked if the clinicians discussed (a) the possibility of a hereditary cancer syndrome (yes/no/don’t know) and (b) related genetic topics. The questionnaire was self-developed by a medical psychologist (K.D.) and clinical geneticist (C.A.) specifically for this study, based on daily practice, and pilot tested among several patients visiting the GIOCA clinic prior to the start of the study.

#### Audiotapes

The audiotaped consultations were transcribed verbatim. To determine whether cancer genetic topics were adequately discussed to guarantee optimal referral for genetic counselling, K.D and C.A. formulated criteria (see Table 2) based on the Dutch CBO-guidelines (http://oncoline.nl/erfelijke-darmkanker). This led to three categories which we visualised as a ‘traffic light’): (1) red; cancer in the family was not discussed, (2) orange; cancer in the family was inadequately discussed, (3) green, cancer in the family was adequately discussed to determine whether an indication for referral to genetic counselling was present.

**Table 2:** Coding scheme used in this study

Traffic light	Category		Description of the content of the audiotaped consultation	Example
	Cancer in the family was	Cancer in the family?		
Green	Adequately discussed	Yes	Discussed are: number of family members with cancer, type of cancer and age. A clear distinction was made between first and second-degree relatives	Do you have other family members with cancer? What type of cancer did they get? How old were they when they got cancer?
		No	Family in the cancer is clearly discussed and there are no other cancers in this family	Do you have other family members with cancer? (and then ask probing questions, such as: Also, no second-degree family members?)
Orange	Inadequately discussed	Yes	The discussion does not fulfil the criteria mentioned above. e.g. the patient gives information about 1 person, and the specialist does not ask about the rest of the family	“How old is your mother?” (Instead of asking how old the family member was at the time of diagnosis)
		No	The discussion is multi-interpretable, therefore it is unclear whether other family members have cancer	“Are there people in your family with cancer or polyps or that kind of thing?”
Red	Not discussed	n/a	The clinician does not ask about cancer in the family	n/a

In addition, we explored the type of questions used by clinicians during consultations in which cancer in the family was inadequately discussed, and coded them with labels such as ‘vague’, ‘multi-interpretable’, and ‘steering’.

#### Medical records

Medical records were reviewed for the following information: (1) if the checklist: (a) was present (yes/no), (b) was complete (yes/no), (c) revealed an indication for referral for genetic counselling based on the available information (yes/no), and (2) if the patient was referred for genetic counselling (yes/no). A checklist was coded as incomplete if one or more of the boxes remained unchecked, or when a question mark was placed next to an uncrossed box.

### Data analysis

Descriptive statistics were used to describe the study sample. Independent t-tests and Chi square tests were used to compare age and gender between the Before and After group. If differences in age and gender were present, logistic regression analysis was used to evaluate whether age and/or gender were significantly related to the outcome variables, i.e. discussing a family history of cancer during the consultation, initiative for discussion, and what was discussed (who, first-degree, second-degree, type and age) at the univariate level. If age and/or gender appeared to have no significant influence, results of the Pearson’s Chi square test and Fisher’s exact test are reported to describe differences between the Before and After group. Otherwise, the results of the logistic regression analyses controlling for age and/or gender are presented.

All analyses were performed using IBM SPSS version 20.0. A *p* value of 0.05 (two-sided) was considered significant.

Using the criteria described above, all transcripts of the audiotapes were reviewed by K.D. and double coded by C.A. Differences between codings were discussed and, in case the coders were uncertain about the coding, a third coder, E.D., was asked to also code the transcripts.

## Results

### Study sample

Table 3 presents the characteristics of the study sample. Most patients were seen by two of the five gastroenterologists in training, or by the surgeon.

**Table 3:** Characteristics of the population sample

Variable	Before (n = 44)		After (n = 52)		*p* value
	Mean	Range	Mean	Range	
Age in years	67.9	43–91	62.6	39–86	0.02

	N	%	N	%	
Gender					0.68
Male	27	61	34	65	
Female	17	39	18	35	
Intake consultation with…					0.74
Gastroenterologist	26	59	29	56	
Surgeon	18	41	23	44	
Participation*					
Questionnaire	44		16	31	
Audiotape	n/a		15	29	
Both audiotape and questionnaire	n/a		21	40	

* Because patients were invited by the researcher for permission to audiotape *before* the consultation, and by the nurse to complete the questionnaire *after* the consultation, some patients did not participate in both audiotaping and the questionnaire

**Fig. 1:**
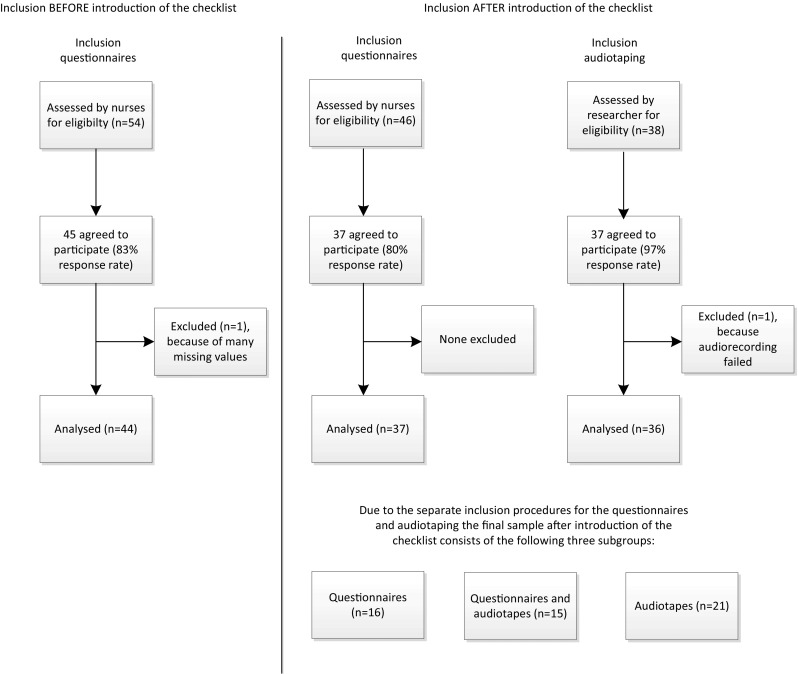
Flow diagram of the inclusion

A flow diagram of the inclusion is show in Fig. 1. In the Before group, 54 patients were eligible of which 45 (83 %) participated. One questionnaire was excluded because of many missing values, resulting in a final sample of 44 patients.

In the After group, 46 eligible patients were approached for the questionnaire and 37 (80 %) participated. In the After group, 38 patients were approached for audiotaping of their consultations, of whom 37 consented (response 97 %); however, because one recording failed, the final sample consisted of 36 audiotapes. Also, because patients were invited by the researcher about audiotaping *before* the consultation, and by the nurse regarding the questionnaire *after* the consultation, some patients did not participate in both audiotaping and the questionnaire (Table 3). In total 52 patients were included in the After group (16 questionnaire only, 15 audiotapes only and 21 both questionnaire and audiotape).

Between the Before and After group there were no significant differences for gender, but patients in the Before group were significantly older (*p* = 0.02). However, as age had no significant influence on any outcome variable, we did not correct for age in the analyses and report the results of the Pearson’s Chi square and Fisher’s exact tests.

#### Did introduction of the checklist increase discussion of family history and genetic counselling?

There were no significant differences between the Before and After group regarding patients’ perception of discussing cancer in the family, but some trends were observed (Table 4). More patients in the After group reported discussing (1) *cancer in the family* (before 77 % vs after 89 %; *p* = 0.16), (2) *second*-*degree family members* (before 53 % vs after 75 %, *p* = 0.15), (3) *type of cancer* (before 75 % vs after 91 %; *p* = 0.24), and (4) *age* at which family members had cancer (before 55 % vs after 76 %; *p* = 0.15). Table 5 shows which topics were discussed with patients who were told by the clinician that there was a suspicion of a hereditary form of cancer.

**Table 4:** Discussion of cancer genetic topics

Topics discussed according to the patients	Before (n = 44)		After (n = 36)		Χ^2^ (*df*)	*p* value
	N	%	N	%		
1. Family members with cancer	34	77	33	89	2.00 (1)	0.16
Patient has no family members with cancer	12	27	11	30		
Patient has family members with cancer	22	50	22	60		
2. Who have had cancer	20	95	21	100	n/a	1.00
3. First-degree family members	19	95	21	96	n/a	1.00
4. Second-degree family members	10	53	15	75	2.12 (1)	0.15
5. Type of cancer of family members	15	75	19	91	n/a	0.24
6. Age at which family members got cancer	11	55	16	76	2.05 (1)	0.15
7. Clinician took the initiative to discuss family history of cancer	29	91	26	84	n/a	0.47

For question 2, 3, 5 and 7 the *p* value from the Fisher’s exact test (2-sided) is reported; the remaining *p* values are from the Pearson’s Chi square test

**Table 5:** Topics discussed in case of possible hereditary cancer

According to patients	Before		After		Χ^2^ (*df*)	*p* value
	N	%	N	%		
The consultation showed that the cancer is possibly hereditary	5	24	7	33	0.47 (1)	0.50
*Topics discussed*						
Why the doctor thinks there is a possibility of hereditary cancer	2	40	4	57		
Which types of genetic tests are available	2	40	4	57		
How genetic testing works	1	20	0	0		
The consequences of genetic testing for the patient self	1	20	0	0		
The consequences of genetic testing for the patient’s family	1	20	2	29		
Something else*	0	0	2	29		
None of these topics were discussed with me	2	40	1	14		

* Advice given to sister, and explanation of statistically high risks

#### Was the quality of discussing family history sufficient to guarantee optimal referral for genetic counselling?

In 28 % (n = 10) of the audiotaped consultations family history was adequately enough discussed to determine whether an indication for referral to genetic counselling was present, in 58 % (n = 21) the discussion was considered inadequate, and in 14 % (n = 5) of the consultations it was not discussed at all. Below, we present examples of adequate and inadequate discussions.

An example of an *adequate* discussion:

Gastroenterologist (G) with female patient

- G: … and I also wondered if, in your family, are there any, do you have **brothers, sisters or parents or grandmother** with **bowel problems**?
  - Patient: My brother had.. that started with esophageal cancer and lung cancer.
- G: Oh.
  - P: And then he got a stoma and the connection was made from out of the stomach
- G: Yes, yes, he had surgery
  - P: in the end that broke down. So he passed away. And my mother had Parkinson’s disease. And my father…[not understandable].
- G: **Alright, but no colorectal cancer or, and also no grandfathers and grandmothers, cousins as far as you are aware**?
  - P: … not the intestines.
- G: … **not the intestines. Or other?** So only your brother had esophageal cancer, but overall not many others.
  - P: … he smoked a terrible amount and also drank a terrible amount.
- G: **But as far as you are aware it is not known whether there are many other tumors, breast cancer, cervical cancer in your family.**
  - P: … my sister has rheumatism, that is also serious, but well, that has nothing to do with it.

An example of an *inadequate* discussion:

Surgeon (S) has a consultation with a male patient.

- S: What I was wondering? **Are there any family members with bowel polyps or colorectal cancer**?
  - P: No.
- S: No. No, nobody?
  - P: No, no. On my mother’s side they are strong.
- S: All right.
- Surgeon is typing.

Another example of an *inadequate* discussion:

Gastroenterologist (G) with male patient

- G: … a**nd are there other family members with it, brothers, sisters or eh.. parents**?
  - Wife: No colorectal cancer, but eh, lung cancer and breast cancer, Hodgkin’s…
  - P: Bladder cancer.
  - Wife: Bladder cancer. Large family. He comes from a large family.
- G: **Yes. But no colorectal cancer?**
  - Wife: No, not as far as we know. No, nobody has it.
- G: All right.

In addition, more in-depth explorative analysis of the audiotapes showed that, in consultations in which cancer in the family was inadequately discussed, the clinicians asked vague, unfinished, very general and steering questions, or more than one question at the same time. Vague questions are unclear questions which could be interpreted in several ways by a patient. For example, ‘and do you have brothers and sisters of your own? All?’. Unfinished questions are questions that need to be filled in by patients themselves and can therefore be interpreted in several ways. For example, ‘No, and do you have sisters with….’. Very general questions are questions that do not direct the patient in any way to become more specific about their family history. For example, ‘What we want to know in advance, thinking it might be cancer, is that we always want to know the family history.’ Steering questions are questions which steer the answer of the patient in a specific direction. For example, ‘and furthermore, what I would like to know, in your family there is nobody with colorectal disease?’. Sometimes clinicians asked several questions at once, As a result, it remains unclear to which part of the question a yes/no answer of the patient refers. For example, ‘but there are not many or other tumours, breast cancer, uterine cancer in your family?”

#### Was the checklist filled in correctly?

Of the 52 medical files of the After group that were reviewed, a checklist was present in 27 (53 %) of them (one file could not be reviewed due to a missing patient number). Of the 27 available checklists, 5 (19 %) were incomplete (e.g. question marks on the checklist, or several boxes not crossed); the remaining 22 (81 %) were completed correctly.

#### Were referrals made when appropriate?

For 6 (29 %) patients the checklist indicated that a referral for genetic counselling would be appropriate. According to their medical files, 5 (83 %) of those patients were referred for genetic counselling and 1 patient (17 %) was not.

## Discussion

This explorative study investigated the role of surgeons and gastroenterologists in training in recognising familial risk for CRC. Also examined was the feasibility of using a checklist to enhance discussion of family history of cancer and referral for genetic counselling.

Contrary to our expectations, the checklist did not increase the discussion of cancer in the family during the first consultation, as reported by the patients. Importantly, cancer in the family was already discussed in most (77 %) of the intake consultations before introduction of the checklist. This relatively high frequency of discussion of cancer in the family (e.g. who have had cancer, type of cancer) suggests a ceiling effect, i.e. little room is left for improvement. Interestingly, two other studies (performed at the same time as ours) demonstrated that oncologists, surgeons and gastroenterologists discussed a cancer family history in a comparable percentage (80 %) of consultations [6, 7]. However, those studies did not explore the detailed content of the discussion of family history during an actual consultation.

When examining details of the discussion on family history of cancer, our study shows that the quality of the discussion is inadequate in most consultations (72 %). Importantly, all but one of the clinicians were residents who, because still in training, may have a harder time recognizing potential at risk patients and discussing family history. However, as they are supervised at distance, investigating family history is an important task as they have to inform their supervisor adequately about the cancer family history of the patient. As a consequence, patients with an indication for genetic counselling could well have been missed. Thus, specific training of communication skills might be warranted. Education of healthcare professionals should not only enable understanding and utilisation, but also effective communication about genetics [12, 13], i.e. investigating the family history in a structured way using open-ended non-steering questions and prompting. In that way, gastroenterologists and surgeons (in training) may better contribute to a more adequate referral of patients to genetic counselling.

In this study, only in about half of the consultations was the checklist present in the medical file and about one-fifth of the checklists were incomplete; this suggests that the clinicians had difficulty using them, or had missed relevant data. The brief introduction given by a clinical geneticist for this study might not have been sufficient for these clinicians. Furthermore, although the checklist was placed on the desk of the health professional by the nurse *before* the consultation, there was no obligation to use it. Also, because health professionals may lack knowledge on genetics [8, 14, 15], clinicians in the present study may have found it difficult to use the checklist properly. In line with our findings, a recent randomised controlled trial among gastroenterologists and surgeons aimed at improving recognition and referral for familial CRC risk (through a website, patient-targeted brochures, and clinician-targeted education and pocket referral cards), showed that improving knowledge did not improve referral rates [9]. Therefore there seems to be potential to improve genetic cancer history taking of residents by adding a mandatory curriculum that addresses proper genetic history evaluation.

Lack of time might also play a role in busy clinics, where diagnosis and treatment planning take place on the same day. Guidelines recommend that familial risk of CRC and preventive measures be assessed on diagnosing CRC (http://oncoline.nl/erfelijke-darmkanker). However, health professionals might not consider this to be the right time to discuss cancer in the family. For 13 patients we explored this further by audiotaping all their consultations during that day. We observed that if this topic was not addressed during the intake consultation, then it was not raised in any of the subsequent consultations on that day either.

Why should we pursue family history at all now DNA technology is readily available at increasingly lower cost [16]? Potentially, all tumours could be tested for Lynch syndrome and the polyposis syndromes. However, if no mutation is detected, surveillance by colonoscopy may still be warranted because the family history might be suggestive for familial CRC. Furthermore, another hereditary condition may be present, such as serrated polyposis, Cowden or Peutz-Jeghers syndrome, and also for these conditions family history may be helpful in revealing these conditions.

It should be noted that this was an explorative study with an uncontrolled before and after design. Therefore, our results might be explained by differences between the groups before and after introduction of the checklist (as is the case for age), and by time effects. Furthermore, as mentioned before there could have been a ceiling effect, because cancer in the family was already discussed in most intake consultations. Also, the checklist was only briefly introduced by the clinical geneticist during a multidisciplinary meeting and this might not have been sufficient instruction for the, residents participating in this study. A strength of the study is the use of a mixed method design, which gives a better impression of what actually happens during a consultation from multiple perspectives. Furthermore, to our knowledge this is the first study to investigate a detailed discussion of the family history in clinical practice.

To conclude, this explorative study shows that gastroenterologists and surgeons, of which most were in training, have difficulty in fulfilling their gatekeeper role of recognising patients at familial risk for CRC. Although they often discuss cancer in the family during the initial consultation, their investigation is not always sufficient to reveal all indications for referral for genetic counselling. Furthermore, this study shows that the introduction of a checklist might not necessarily solve the problem of suboptimal referral. Interventions other than a checklist (e.g. training in communication skills) might be needed to improve exploration of cancer in the patient’s family and improve the referral rates.
